# Case Report: Artery of Percheron infarction following surgical clipping of multiple intracranial aneurysms

**DOI:** 10.3389/fsurg.2025.1623891

**Published:** 2025-07-14

**Authors:** Min Chen, Xiangping Xia, Linhui Chen, Lei Yang, Zhiqi Li, Bin Xu, Feng Xu

**Affiliations:** ^1^ Department of Neurosurgery, Anqing Municipal Hospital, Anqing, Anhui, China; ^2^ Department of Neurosurgery, Affiliated Hospital of Zunyi Medical University, Zunyi, Guizhou, China; ^3^ Department of Neurocritical Care, Huashan Hospital, Shanghai Medical College, Fudan University, Shanghai, China; ^4^ Department of Neurosurgery, Huashan Hospital, Shanghai Medical College, Fudan University, Shanghai, China; ^5^ National Center for Neurological Disorders, Shanghai, China; ^6^ Shanghai Key Laboratory of Brain Function and Restoration and Neural Regeneration, Shanghai, China; ^7^ Neurosurgical Institute of Fudan University, Shanghai, China; ^8^ Shanghai Clinical Medical Center of Neurosurgery, Shanghai, China

**Keywords:** artery of percheron, paramedian thalamic infarction, surgical clipping, multiple intracranial aneurysms, tirofiban, case report

## Abstract

**Background:**

Occlusion of the artery of Percheron (AOP), a rare variant of the paramedian thalamic artery, leads to bilateral paramedian thalamic infarction with or without midbrain involvement. AOP following surgical clipping of anterior circulation aneurysms has not been documented in the literature.

**Case description:**

A 59-year-old female patient presented with recurrent dizziness and diplopia, for which she subsequently received dual antiplatelet therapy. Angiography revealed hypoplasia of the right P1, but identified multiple intracranial aneurysms. The patient underwent one-stage clipping. Twelve hours postoperatively, the patient experienced sudden onset of loss of consciousness. Head CT revealed no hemorrhage or infarctions. Subsequent CT angiography (CTA) showed no large vessel occlusion, and CT perfusion (CTP) indicated no definitive core infarction or hypoperfusion zones. Due to contraindications for thrombolysis following surgery, intravenous tirofiban was administrated as an antiplatelet therapy. MR imaging then demonstrated high signal intensity in the bilateral paramedian thalami without midbrain involvement on DWI. At discharge, the patient recovered normal mental status, but still had mild memory deficit.

**Conclusion:**

For patients with multiple intracranial aneurysms concomitant with vertebrobasilar transient ischemic attacks (TIAs), it is important to be vigilant about the possibility of posterior circulation perforating artery infarction following clipping. For patients presenting with altered consciousness, vertical gaze palsy, and memory impairment, if cranial MRI reveals infarctions in the bilateral paramedian thalamic regions, AOP infarction should be considered first. Raising awareness of AOP infarction, along with early detection, diagnosis, and treatment, can significantly improve clinical symptoms and prognosis for these patients.

## Introduction

The artery of Percheron (AOP) is a rare variant of the paramedian thalamic artery, originating as a single trunk from the P1 segment of one posterior cerebral artery (PCA). It supplies the bilateral paramedian thalami and rostral midbrain (1). Occlusion of the AOP can lead to bilateral paramedian thalamic infarction with or without midbrain involvement, known as AOP infarction. AOP infarction is rare and presents with various clinical manifestations, most commonly characterized by a triad of altered consciousness, vertical gaze palsy, and memory impairment (2–5). AOP following surgical clipping of anterior circulation aneurysms has not been documented in the literature. This study firstly reports a case of AOP infarction following surgical clipping of multiple intracranial aneurysms and discusses the pathogenesis, clinical presentation, imaging features, and treatment of AOP infarction in conjunction with a literature review. The aim is to enhance clinicians' vigilance, thereby facilitating prompt diagnosis and the implementation of appropriate therapeutic interventions.

## Case description

**Figure 1:**
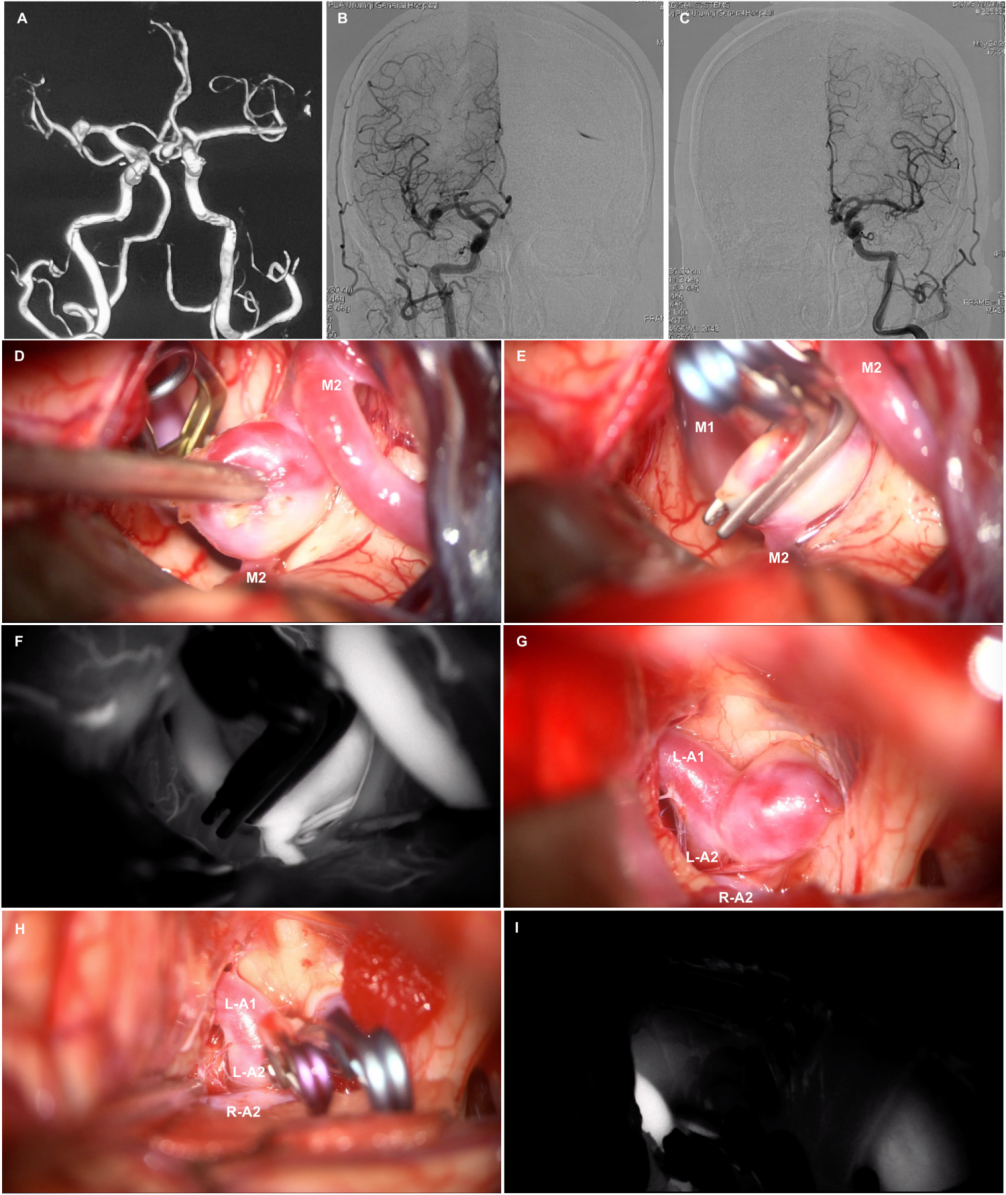
Brain CTA **(A)** reveals multiple intracranial aneurysms in the ACoA. and right MCA bifurcation. Anteroposterior view of the right **(B)** and left **(C)** internal cerebral artery (ICA) angiogram. Intraoperative photos show perpendicular clipping in the right MCA bifurcation aneurysm **(D,E)**, and multiple clipping in the ACoA aneurysm **(G,H)**. Intraoperative indocyanine green angiography confirmed complete obliteration of the aneurysm and patency of the parent vessels **(F,I)**.

A 59-year-old female patient presented in June 2023 with a chief complaint of “recurrent dizziness for more than 6 weeks.” Approximately six weeks prior, the patient experienced the onset of dizziness without any precipitating factors. The dizziness was non-vertiginous and was accompanied by diplopia and nausea, but without tinnitus, hearing loss, altered consciousness, or seizure activity. The symptoms abated after several minutes of rest, and she did not seek medical attention at that time. Two weeks later, the dizziness and diplopia recurred intermittently. Magnetic resonance imaging (MRI) demonstrated minor ischemic lacunar lesions in the bilateral frontal and parietal lobes. Considering the patient's history of hypertension and transient diplopia, vertebrobasilar transient ischemia attack was suspected. The patient received dual antiplatelet therapy. Subsequent head computed tomography angiography (CTA) revealed no obvious stenosis of the vertebrobasilar artery, but multiple saccular intracranial aneurysms were detected (Figure 1A). Cerebral angiography demonstrated a 4 mm × 3 mm aneurysm of the anterior communicating artery (ACoA) and a 6 mm × 4 mm aneurysm of the right middle cerebral artery (MCA) bifurcation (Figures 1B,C). The patient was referred to our facility for further evaluation and management. On admission, her blood pressure was 146/88 mmHg. Neurological examination revealed a clear sensorium, equal and reactive pupils, intact light reflex, and appropriate verbal responses. Motor examination showed normal strength (grade V) in all limbs, with no pathological reflexes. Cardiac ultrasound showed no abnormalities**,** and the 24-hour Holter monitoring did not detect arterial fibrillation. Cardiac CT also showed no significant abnormalities in the coronary calcium score, but noted dilation of the ascending aortic root.

After receiving dual antiplatelet therapy, the patient did not experience any further posterior circulation transient ischemic attacks. Considering the increased risk of aneurysm rupture with dual antiplatelet therapy, we opted to first address multiple intracranial aneurysms using one-stage clipping. One week after discontinuing clopidogrel, the patient underwent a right supraorbital lateral approach for clipping of multiple intracranial aneurysms under general anesthesia. Dissection of the sylvian fissure from distal to proximal revealed a saccular aneurysm located at the M1-M2 bifurcation of the right MCA (Figure 1D), with a neck measuring approximately 6 mm in width. Temporary occlusion of the M1 segment was performed, allowing for complete dissection and exposure of the aneurysm. A vertical clip (Yasargil Aneurysm Clip System, FT820T; Aesculap) was applied to occlude the aneurysm neck. Following the release of the temporary clip, a second clip (Yasargil Aneurysm Clip System, FT820T; Aesculap) was positioned in a stacked configuration to secure the residual aneurysm (Figure 1E). The optic and carotid cisterns on the right side were further explored, revealing a saccular aneurysm at the anterior communicating artery that was compressing the optic chiasm (Figure 1G). Temporary occlusion of the left A1 segment was performed, and meticulous dissection of the aneurysm neck was followed by the application of a straight clip (Yasargil Aneurysm Clip System, FT960T; Aesculap) to the neck, with a mini curved clip (Yasargil Aneurysm Clip System, FT712T; Aesculap) applied to the residual neck portion (Figure 1H). Following the release of the temporary clip, intraoperative indocyanine green angiography confirmed complete obliteration of the aneurysm and patency of the parent vessels (Figures 1F,I). Intraoperative electrophysiological monitoring showed no decline in motor or somatosensory evoked potentials. Postoperatively, the patient regained consciousness with preserved speech and motor functions of the extremities.

**Figure 2:**
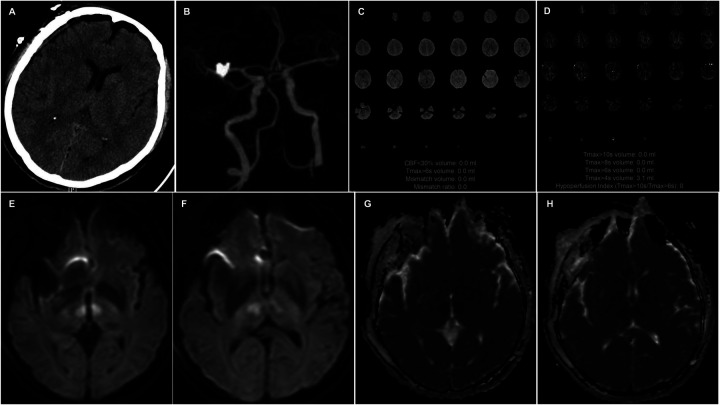
A noncontrast head CT **(A)** scan reveals no acute hemorrhage or infarctions. CTA **(B)** shows no large vessel occlusion, and CTP **(C,D)** indicates no definitive core infarction or hypoperfusion zones. Axial DWI **(E,F)** images show high signal intensity in the bilateral paramedian thalami, with corresponding decreased apparent diffusion coefficient (ADC) values **(G,H)**.

**Figure 3:**
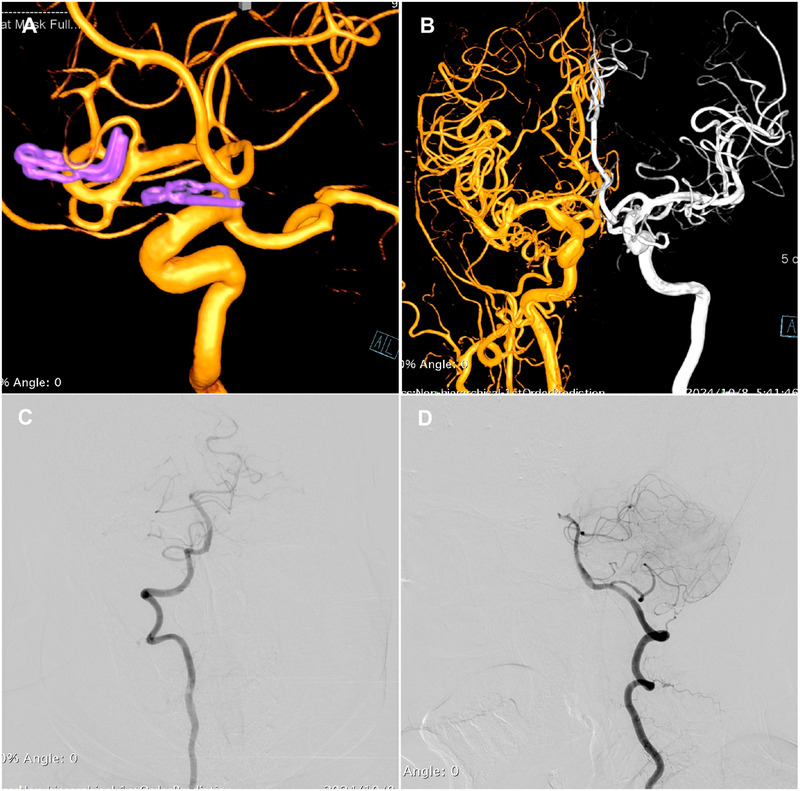
Three-dimensional DSA images of the right ICA **(A)** demonstrate no residual or recurrence of the aneurysm. **(B)** Fusion of bilateral ICA angiography. VA angiograms show hypoplasia of the right P1 segment of the PCA and no visualization of the AOP **(C,D)**.

**Figure 4:**
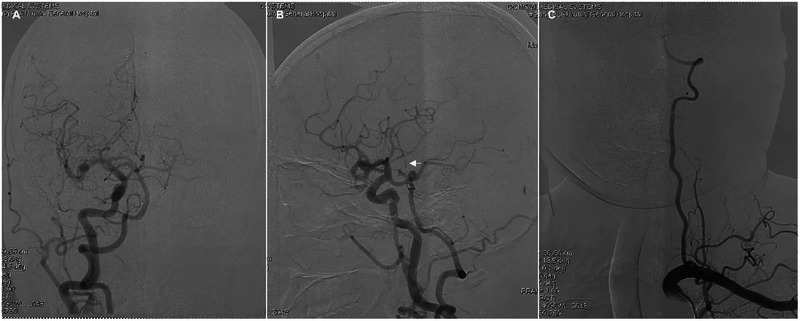
Preoperative angiograms show unilateral complete fetal PCA on the right side **(A,B)** and hypoplasia of the left VA **(C)**. Arrow indicates the thalamic perforating artery (i.e., an AOP).

Twelve hours postoperatively, the patient experienced sudden onset of loss of consciousness, becoming unresponsive, without any convulsive movements. Emergency examination indicated a state of stupor, with bilateral pupils measuring 2 mm and exhibiting sluggish photoreactivity. The nasolabial folds were symmetrical, and the gaze was fixed, with no nuchal rigidity observed. The left extremities exhibited reduced voluntary movement, while the right extremities localized to painful stimuli, and bilateral Babinski signs were positive. A noncontrast head CT scan revealed no acute hemorrhage or infarctions (Figure 2A). An expedited in-hospital stroke protocol was initiated, including a one-stop CTA and CT perfusion (CTP). Cranial CTA (Figure 2B) showed no large vessel occlusion, and CTP (Figures 2C,D) indicated no definitive core infarction or hypoperfusion zones. The possibility of acute infarction in the perforator territory of the posterior circulation was considered. Due to contraindications for thrombolysis following multiple intracranial aneurysm clippings, intravenous tirofiban was administered. Twelve hours post-event, MRI demonstrated high signal intensity in the bilateral paramedian thalami on diffusion-weighted imaging (DWI) (Figures 2E,F), with corresponding decreased apparent diffusion coefficient (ADC) values (Figures 2G,H). The patient was managed with intubation, mechanical ventilation, volume expansion, vasospasm prophylaxis, and neuroprotective strategies. By postoperative day 3, the patient's consciousness had improved, allowing for extubation, and her condition gradually stabilized. At discharge, the patient was alert, with partial memory impairment, normal muscle strength and tone in the limbs, and bilateral positive Babinski signs. One year postoperatively, follow-up digital subtraction angiography (DSA) demonstrated no residual or recurrent aneurysms at the right MCA bifurcation and ACoA (Figures 3A,B). Vertebral artery (VA) angiograms showed hypoplasia of the right P1 segment of the PCA and no visualization of the AOP (Figures 3C,D), which might be present in preoperative images (Figure 4).

## Discussion

The thalamus is primarily divided into four vascular territories: the anterior, posterior, paramedian, and inferolateral regions (6). These are supplied by the polar artery (also known as the thalamotuberal artery), the posterior choroidal artery, the thalamic paramedian artery (also known as the thalamoperforating artery), and the thalamogeniculate artery, respectively. The thalamic paramedian artery, originating from the P1 segment of the PCA, exhibits considerable anatomical variation. According to the study by Percheron et al. (1), there are four main anatomical types. Type I is the most common, where the paramedian arteries originate separately from the proximal segments of the left and right PCAs, each supplying the ipsilateral paramedian thalamus. In Type IIa, both paramedian arteries arise from the P1 segment of either the left or right PCA and supply the bilateral paramedian thalamic regions. Type IIb, also known as the Percheron artery, involves a single artery originating from the P1 segment of one PCA, bifurcating to supply both the paramedian thalamus and rostral midbrain. Type III is characterized by the presence of a communicating artery between the proximal P1 segments of the left and right PCAs, with the paramedian artery originating from this communicating vessel.

AOP infarction is relatively uncommon, accounting for approximately 0.1%–2% of all ischemic strokes (7–9) and 4%–18% of all thalamic infarctions (2, 6, 9). The pathogenesis of AOP infarction primarily involves small vessel disease, cardioembolic events, and large artery disease. Cardioembolic sources include atrial fibrillation, patent foramen ovale, ventricular aneurysm, and valvular heart disease (10–12). Other etiologies encompass hemodynamic changes, hypercoagulable states, vasospasm, and vasculitis (4). Risk factors comprise hypertension, diabetes mellitus, hyperlipidemia, smoking, cerebral atherosclerosis, transient ischemic attacks, and cardiac disorders. During surgical clipping of the ACom and MCA bifurcation aneurysms, temporary occlusion of the A1 or M1 segment was performed. The occlusion was done only once and lasted less than 3 min. If any perforator-related events occur, they will not involve infarction of the posterior circulation perforators. Therefore, aneurysm clipping was not directly related to infarction of the Percheron artery. We thought the AOP infarction was associated with previous vertebrobasilar TIAs. After receiving dual antiplatelet therapy, the patient did not experience any further posterior circulation transient ischemic attacks. Subsequent angiography revealed no obvious stenosis of the vertebrobasilar artery, but multiple intracranial aneurysms were detected. Considering the increased risk of aneurysm rupture with dual antiplatelet therapy, we opted to first address multiple intracranial aneurysms. One week after discontinuing clopidogrel, the patient underwent one-stage clipping of multiple intracranial aneurysms. Therefore, we thought the cause of AOP infarction after surgery might be incomplete antiplatelet treatment.

Cardiac echocardiography showed no abnormalities, and the 24-hour Holter monitoring did not detect arterial fibrillation, ruling out the possibility of cardiogenic embolism. Previous studies suggest that a fetal-type PCA on one side may be a potential congenital variant predisposing to AOP infarction (3, 13). It is hypothesized that when one PCA originates from the ipsilateral internal carotid artery, the P1 segment of the contralateral PCA gives rise to the Percheron artery, supplying both thalami. Hypoplastic or absent P1 segments were more likely to have isolated bilateral paramedian thalamic lesions. In this case, hypoplasia of the right P1 was also detected. Considering the patient's history of hypertension, episodes of posterior circulation TIAs, and lacunar infarcts visible on MRI, it suggested that small vessel disease might be the main cause.

AOP infarction typically presents with an acute onset, with primary clinical manifestations including varying degrees of altered level of consciousness (such as somnolence, stupor, and coma), oculomotor disturbances (including vertical gaze palsy, oculomotor nerve palsy, pseudo-abducens nerve palsy, and pupillary changes), and memory impairment. Additional symptoms may encompass dysarthria, behavioral changes, motor disturbances, thalamic dementia, and mutism (2, 3, 14–16). These clinical features are closely related to the affected anatomical regions. Altered level of consciousness is associated with damage to the intralaminar nuclei of the thalamus, affecting the ascending reticular activating system in the midbrain. Vertical gaze palsy is linked to impairment of the medial longitudinal fasciculus in the midbrain, while memory deficits are often related to damage to the dorsomedial nucleus and limbic fibers involving the thalamus. In this case report, bilateral paramedian thalami were involved, with acute onset presenting as altered consciousness, making it challenging to detect vertical gaze palsy and memory impairment initially. The absence of midbrain infarction resulted in no significant vertical gaze palsy at discharge, although memory impairment persisted.

MRI plays a pivotal role in the diagnosis of Percheron artery infarction. The characteristic imaging features include bilateral paramedian thalamic paired butterfly-shaped lesions with long T1 and T2 signals, and hyperintensity on diffusion-weighted imaging (DWI) during the acute phase. The “V-sign” on Flair and DWI sequences is observed in 67% of Percheron artery infarctions that are accompanied by midbrain infarction, with the midbrain “V-sign” being particularly diagnostic (2). According to Lazzaro et al. (2), AOP infarctions can be categorized into four types based on MRI findings: Type I involves the bilateral paramedian thalami and midbrain; Type II affects only the bilateral paramedian thalami without midbrain involvement; Type III includes the bilateral paramedian thalami, anterior thalami, and midbrain; and Type IV involves the bilateral paramedian thalami and anterior thalami but spares the midbrain. The patient in this case represents Type II, as DWI showed hyperintensity in the bilateral paramedian thalami without midbrain involvement. Angiograms revealed a right fetal-type PCA, suggesting that the event might be associated with AOP occlusion.

The treatment of acute AOP infarction is similar to other types of infarctions, including thrombolysis, antiplatelet aggregation, stabilizing plaque by regulating lipids, improving cerebral circulation, and nourishing nerves. Venous thrombolysis can significantly improve the prognosis of patients with AOP infarction, reducing neurological deficits and sequelae (17). Kostanian (12) reported a case in which angiography showed reperfusion of AOP after superselective arterial thrombolysis in the acute phase, with a significant improvement in neurological deficits. Due to the complex clinical presentation of AOP infarction, early negative findings on cranial CT, in most cases, a definitive diagnosis can only be made after MRI examination, missing the thrombolysis time window. In this case, the patient underwent craniotomy and clipping for multiple intracranial aneurysms, considering venous thrombolysis contraindicated, and was treated with clopidogrel for antiplatelet aggregation. In-hospital multimodal CT did not show large vessel occlusion, and no cerebral angiography or superselective arterial thrombolysis was performed. Despite the recovery of this patient without limb paralysis and language impairments, there was a significant decline in memory and cognitive function. These findings imply that presentation of bilateral medial thalamic infarctions, in the absence of basilar artery occlusion, should elevate clinical suspicion for occlusion of the artery of Percheron. This scenario warrants consideration of interventional strategies targeting the occlusion for potential therapeutic intervention (18). Therefore, early identification, diagnosis, and the adoption of proactive and effective treatment measures such as superselective intra-arterial thrombolysis are crucial for AOP infarction.

## Conclusion

In conclusion, for patients with multiple intracranial aneurysms concomitant with vertebrobasilar TIAs, it is important to be vigilant about the possibility of posterior circulation perforating artery infarction following surgical clipping. For patients presenting with altered consciousness, vertical gaze palsy, and memory impairment, if cranial MRI reveals infarctions in the bilateral paramedian thalamic regions, with or without midbrain involvement, AOP infarction should be considered first. It is important to further conduct cerebrovascular imaging, cardiac ultrasound, and arterial ECG to identify the underlying cause. Raising awareness of AOP infarction, along with early detection, diagnosis, and treatment, can significantly improve clinical symptoms and prognosis for these patients.

## Data Availability

The original contributions presented in the study are included in the article/Supplementary Material, further inquiries can be directed to the corresponding authors.
